# Appearance of Uterine Scar Due to Previous Cesarean Section on Hysterosalpingography: Various Shapes, Locations and Sizes

**DOI:** 10.5812/iranjradiol.5143

**Published:** 2013-05-20

**Authors:** Firoozeh Ahmadi, Leila Torbati, Farnaz Akhbari, Gholam Shahrzad

**Affiliations:** 1 Department of Reproductive Imaging at Reproductive Biomedicine Research Center, Royan Institute for Reproductive Biomedicine, ACECR, Tehran, Iran

**Keywords:** Hysterosalpingography, Cesarean Section, Uterus

## Abstract

Hysterosalpingography (HSG) is the radiographic evaluation of the uterus and fallopian tubes that is used predominantly in the assessment of infertility and evaluation of abnormalities of the uterus and fallopian tubes. Some of the abnormalities that can be detected by HSG include congenital anomalies, polyps, leiomyomas, synechiae and adenomyosis. HSG is also used to evaluate any scarring on the uterus and fallopian tubes.

Cesarean section is the most commonly performed surgical procedure involving the uterus in fertile women. Cesarean section involves an incision made in the lower uterine segment or isthmus. Various changes in the site of the cesarean incision may be seen due to wall weakness and fibrosis. The scar may have various shapes; unilateral or bilateral, single or multiple, wedge-shaped or linear. Awareness of the appearance and locations of uterine defects due to previous cesarean section is necessary in order to differentiate them from normal variations and other pathologies mimicking it.

In this study, we demonstrate the appearance of anatomic defects of the uterine cavity on HSG after cesarian section. We define different shapes such as thin linear defect, focal saccular outpouching, unilateral or bilateral diverticula (dog-ear like) and fistula and different locations such as the uterine body, lower uterine segment, uterine isthmus and the upper endocervical canal.

## 1. Introduction

There are different methods and instruments that can be used to perform a hysterosalpingography (HSG) .The procedure must be performed under strict sterile conditions, since the peritoneal cavity can easily become infected, with the infection spreading through the contrast medium. HSG is performed during the proliferative phase, after cessation of menstruation and before ovulation, between days 7 and 11, in order to avoid any early pregnancies (1-3). Furthermore, the endometrium is thin in this time period, so the image obtained may be better interpreted (4, 5).

The patient is placed on a radiographic table in a lithotomy position. After insertion of the speculum, the cervix is grasped with a tenaculum at 12 o’clock and brought forward to straighten the uterus. A Jarco cannula is used to instill the contrast media (Visipaque 320mg/ml) into the cervix. All air bubbles should be removed from the cannula before injection. Under fluoroscopic monitoring, at least four images are obtained routinely, each time by instillation of 2-3 ml of contrast media.

1) Visualization of intrauterine lesions such as small polyps or synechiae may be better achieved when the uterus is partially filled with media.

2) The best time to evaluate the shape of the uterus is when the uterus is filled completely.

3) The appropriate time to check whether the fallopian tubes are obstructed or not are when the fallopian tubes are filled and intraperitoneal spillages are depicted.

4) Delayed image taken 30 minutes after removal of the instrument from the cervix is the choice to rule out peritoneal adhesions.

In patients suffering from chronic pelvic infection or an untreated sexually transmitted disease, some physicians prescribe antibiotics prior to or after the procedure. HSG is not routinely used for evaluation of a cesarean section scar; however, if done, HSG should be postponed to three months after the cesarean section (6, 7). Evaluation of the cesarean scar is performed to choose the technique of future delivery and prevention of uterine rupture and in cases of abnormal bleeding after delivery (6-8).

Accumulation of blood or secretions in the scar leads to unreliable HSG results (7, 9).

**2. Locations of uterine scar in patients with cesarean section history**

2.1. Uterine body (scar in this section is rare) (Figure 1)

**Figure:**
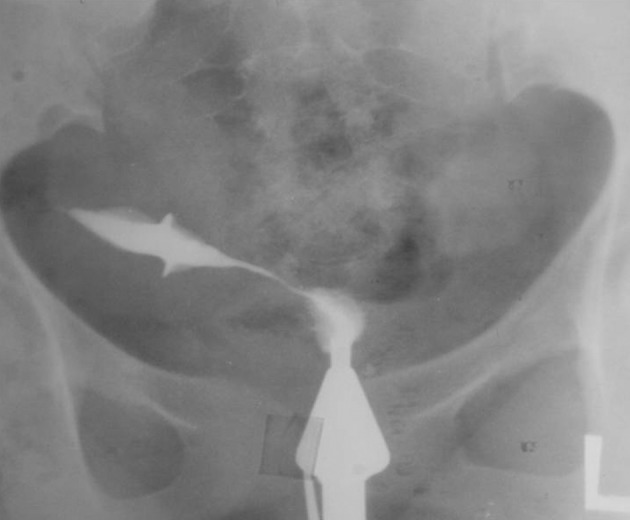
Figure 1. A 25-year-old lady with a history of cesarean section and thin linear defect at the uterine body

2.2. Lower uterine cavity (Figure 2)

**Figure:**
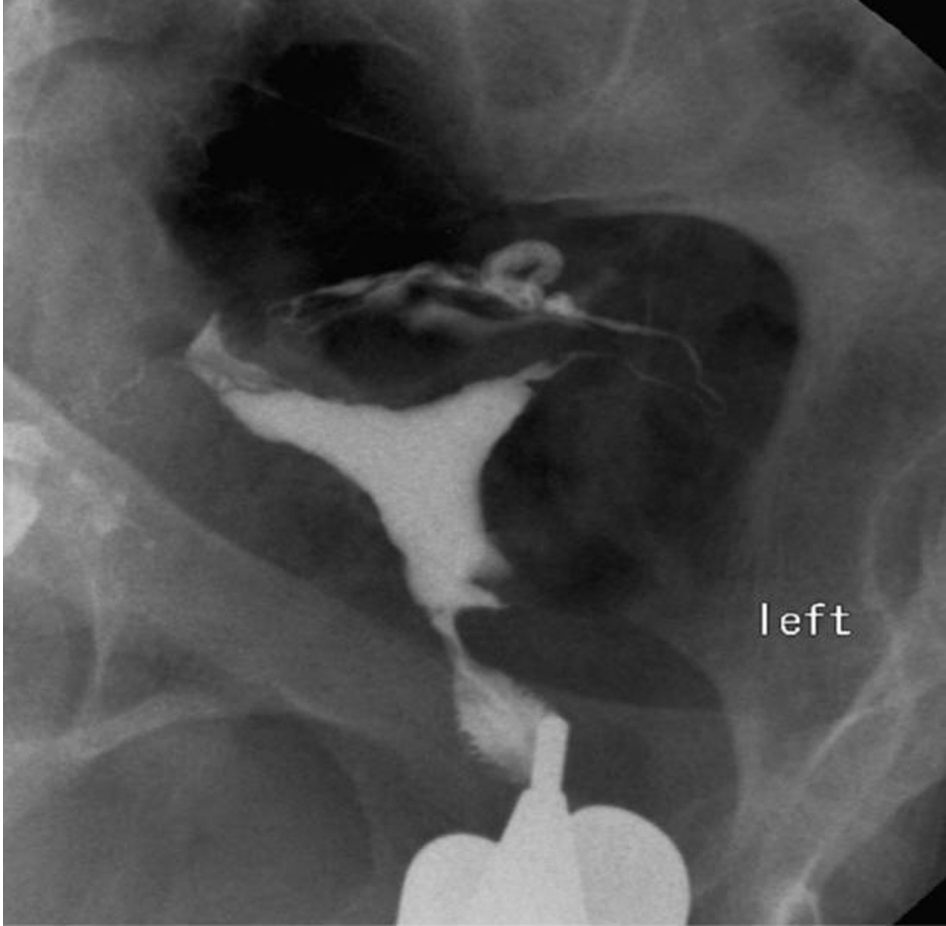
Figure 2. A 31-year-old woman with a previous cesarean section. A wedge-shape defect is seen in the lower uterine cavity.

2.3. Uterine isthmus (Figure 3)

**Figure:**
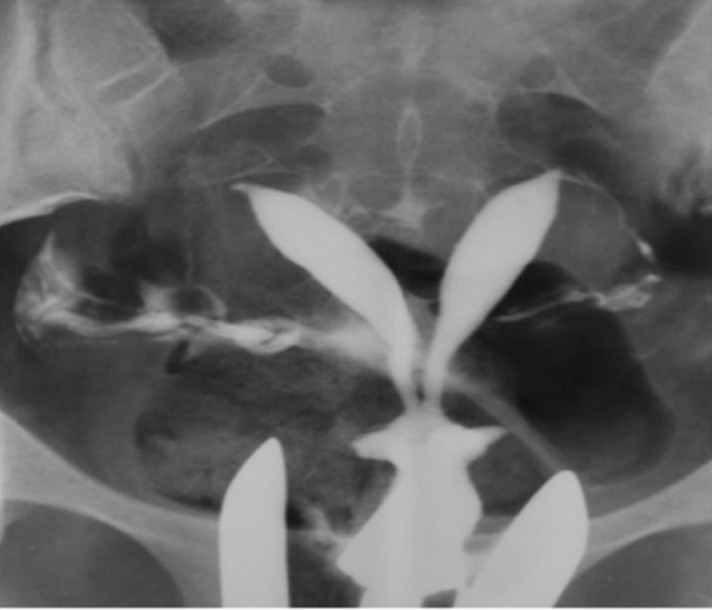
Figure 3. A 28-year-old woman with cesarean scar defect at the isthmus of the long septated uterus

2.4. Upper endocervical canal (Figure 4)

**Figure:**
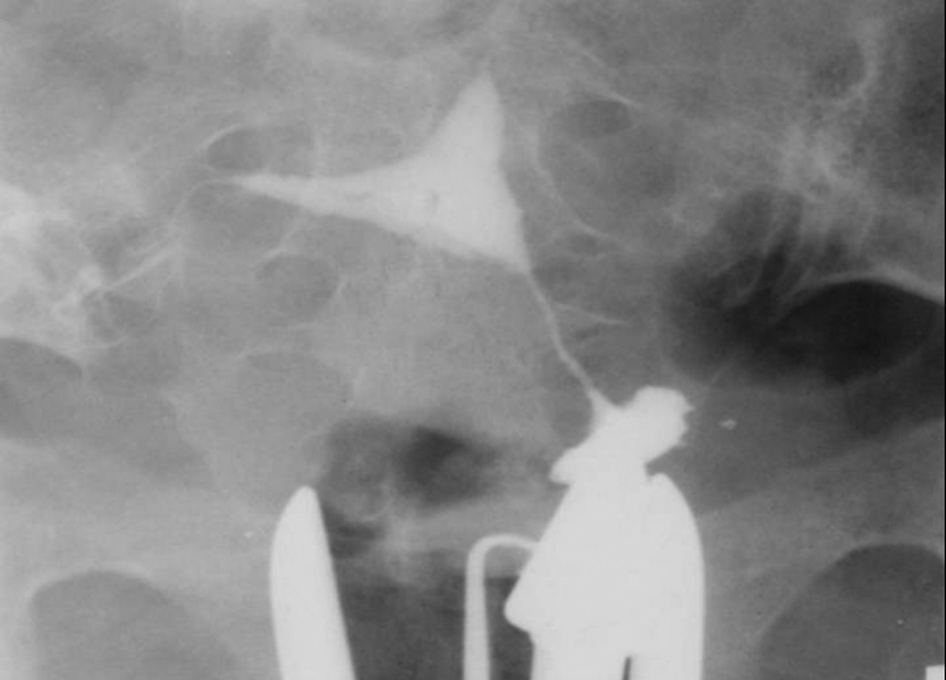
Figure 4. A 28-year-old lady with a large cesarean scar defect at the endocervical canal (outpouching cesarean scar defect)

**3. Shapes of uterine scar in patients with cesarean section history**

3.1. Thin linear defect (Figure 5)

**Figure:**
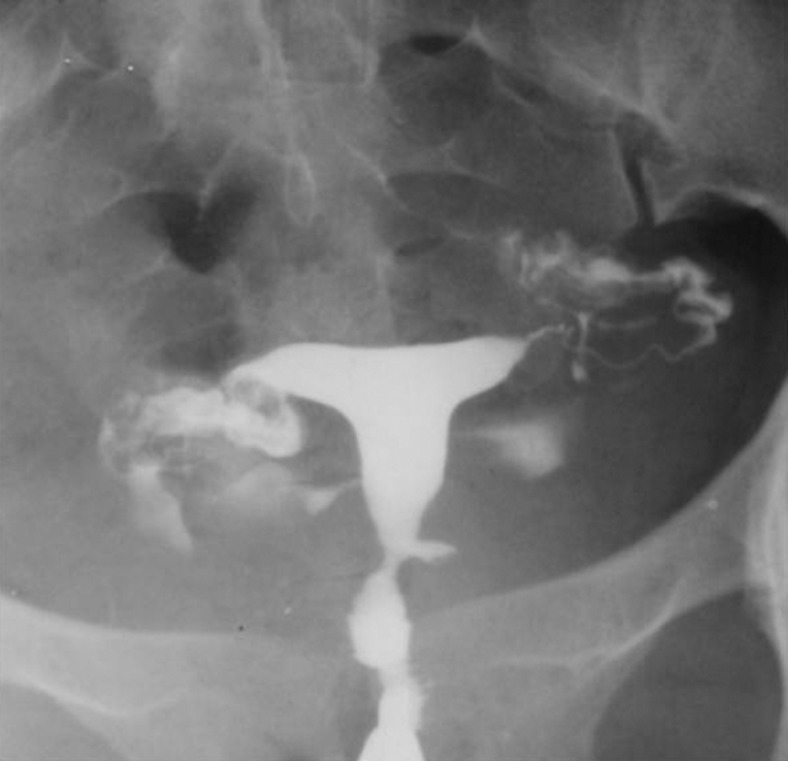
Figure 5. A 29-year-old lady with a thin linear defect at the left lower uterine cavity

3.2. Wedge-shaped defect (triangular) (Figure 6)

**Figure:**
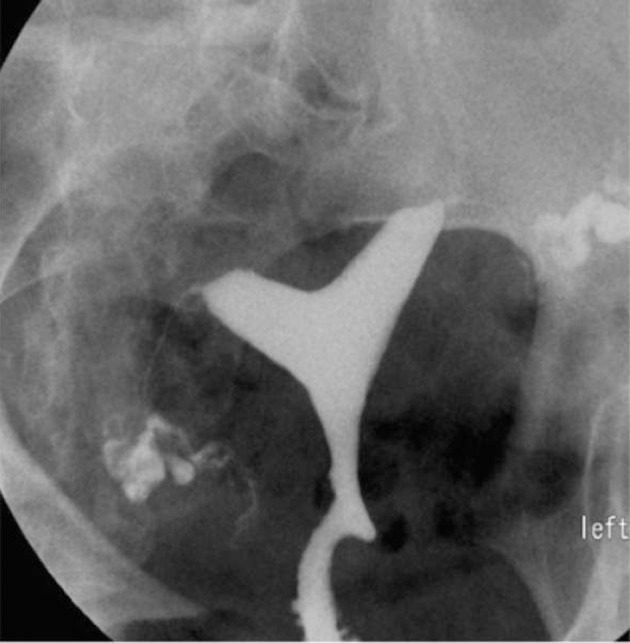
Figure 6. A 29-year-old lady with a wedge-shaped defect (triangular) at the uterine isthmus

3.3. Focal saccular outpouching (Figure 7)

**Figure:**
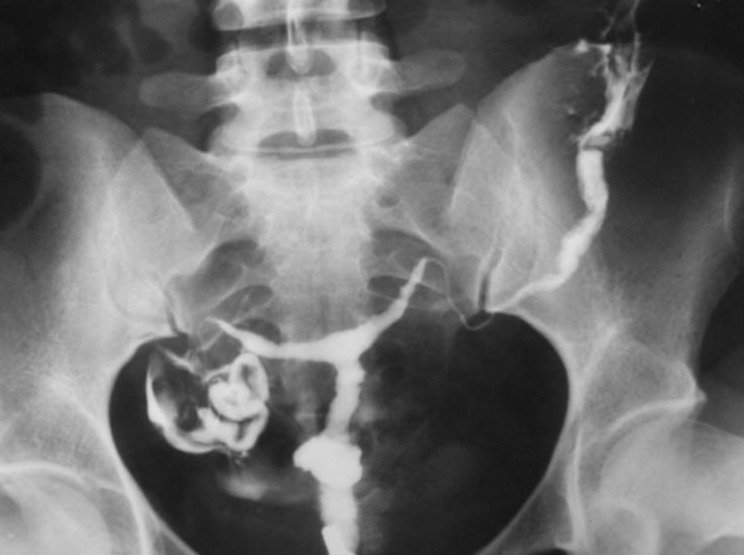
Figure 7. A 27-year-old woman with focal saccular outpouching at the uterine isthmus

3.4. Pseudodiverticula [unilateral or bilateral (dog-ear like)] (Figure 8)

**Figure:**
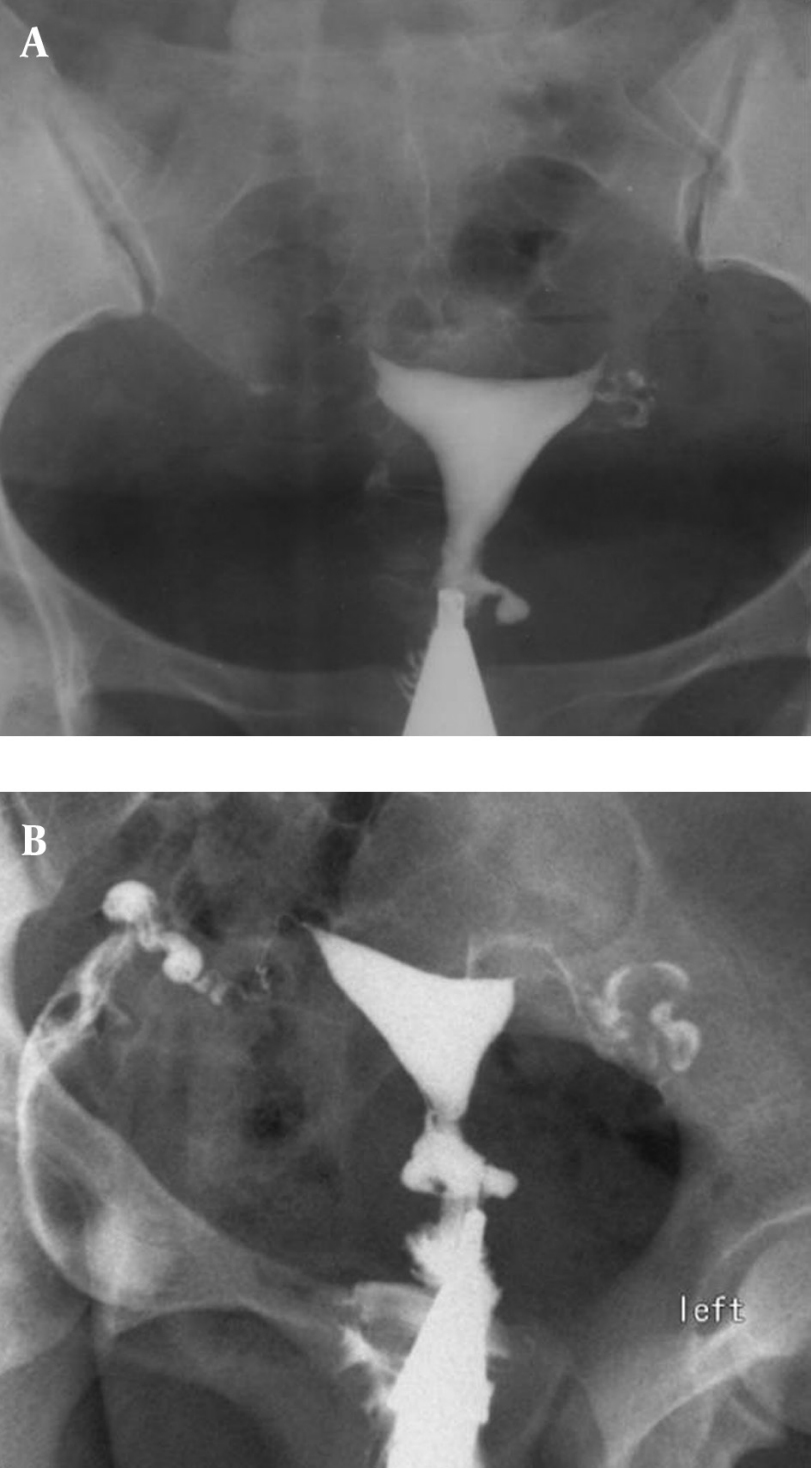
Figure 8. Two 26- and 27-year-old women with (A) unilateral and(B) bilateral pseudodiverticula (dog-ear appearance) at the uterine isthmus

3.5. Overhanging defect that shows a congested endometrium above the scar recess and results from prominence of the superior aspect of the defect and narrowing of its lower border (Figure 9)

**Figure:**
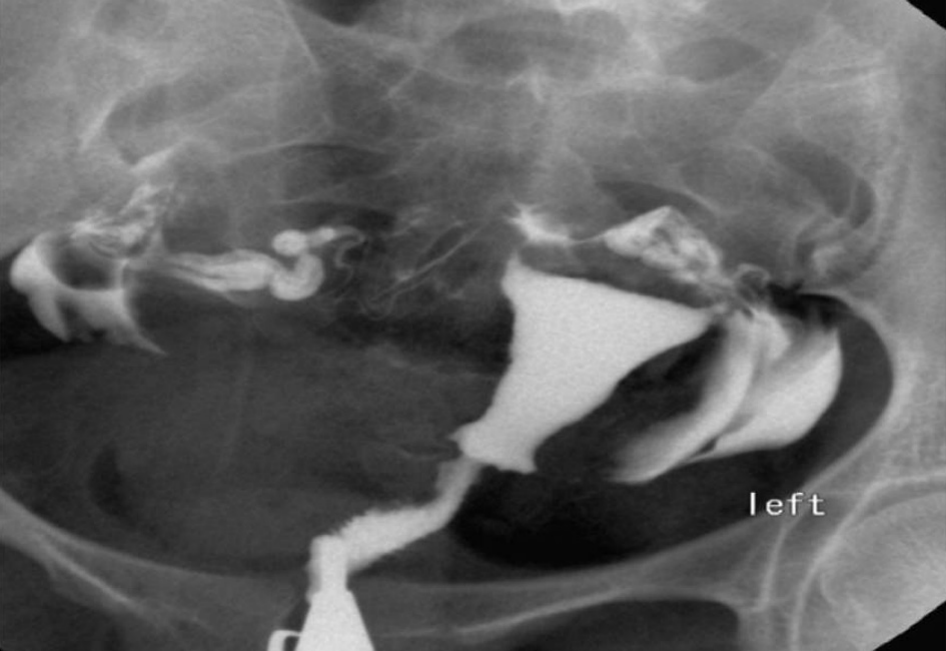
Figure 9. A 32-year-old woman with an overhanging cesarean defect due to prominence of the superior aspect of the defect and narrowing at the uterine isthmus

3.6. Hanging or anchoring defect showing a narrowed region above the scar recess (Figure 10)

**Figure:**
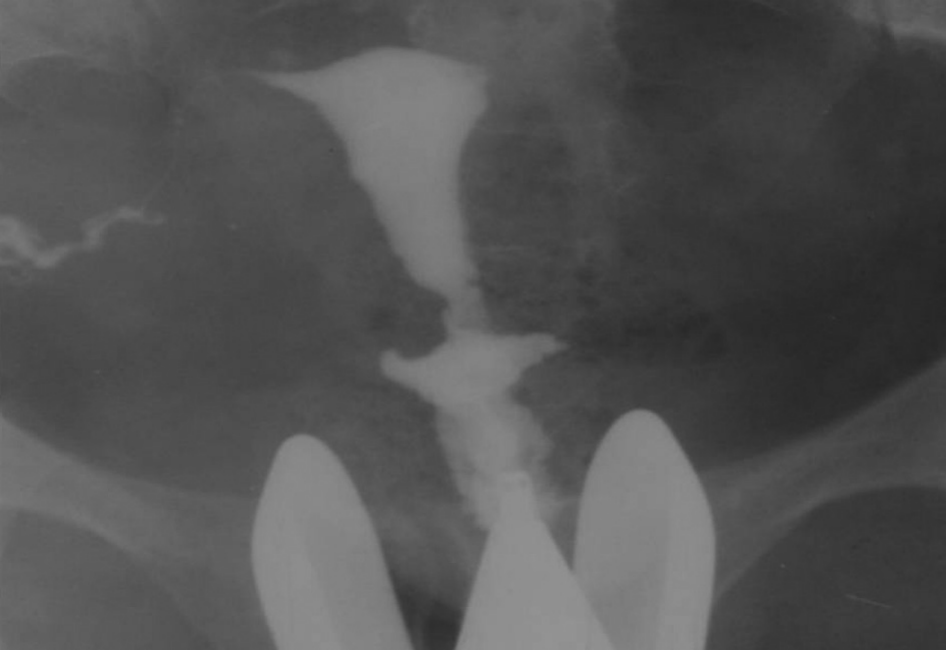
Figure 10. A 32-year-old lady with hanging (anchoring) defect resulting from a narrowed region above the cesarean scar at the uterine isthmus

3.7. Irregularity and narrowing (Figure 11)

**Figure:**
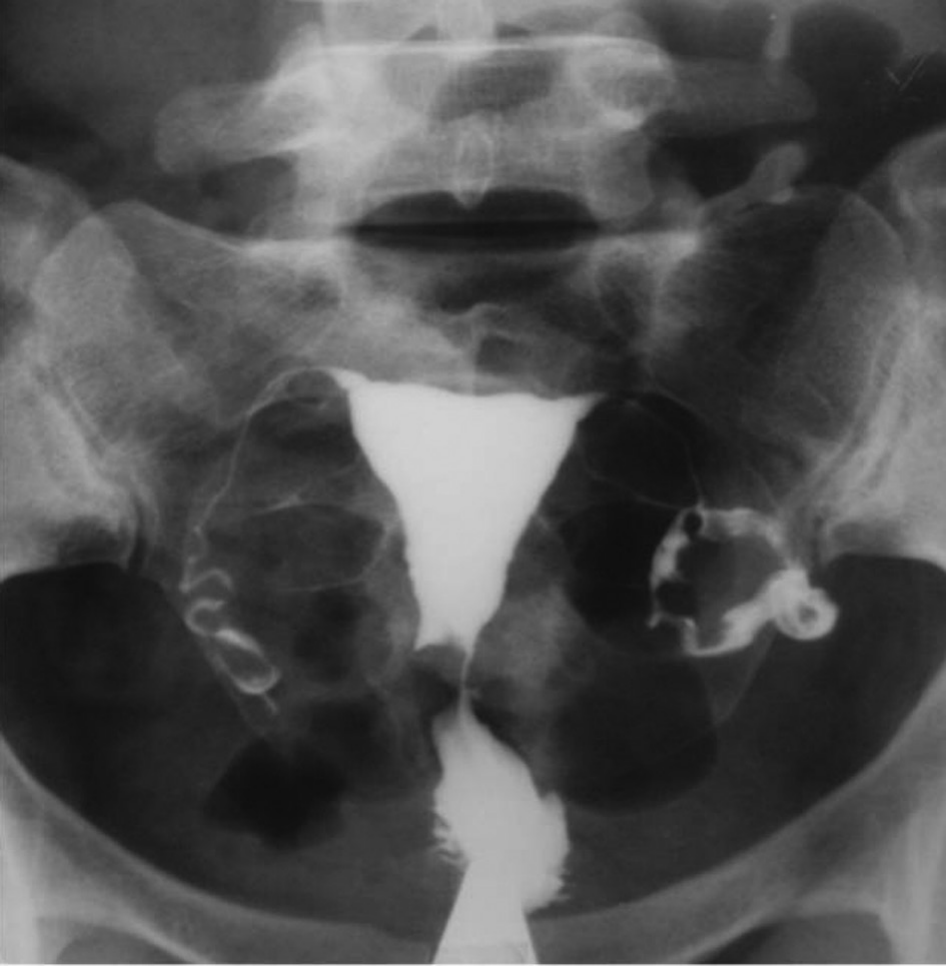
Figure 11. A 33-year-old lady with narrowing and irregularity at the uterine isthmus secondary to cesarean scar

3.8. Atypical insertion in scar recess (Figure 12)

**Figure:**
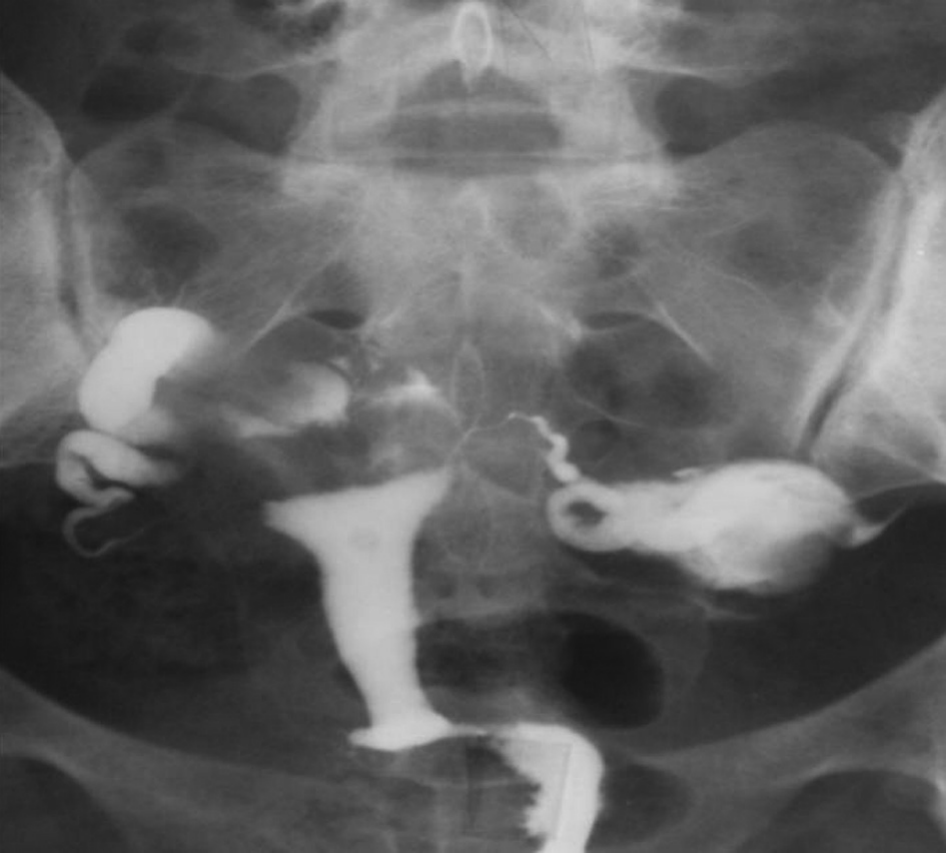
Figure 12. A 32-year-old lady with a typical insertion of the isthmus to the lower uterine cavity secondary to cesarean section

3.9. Fistula to other organs such as the urinary bladder or free soft tissue fistula (Figure 13)

**Figure:**
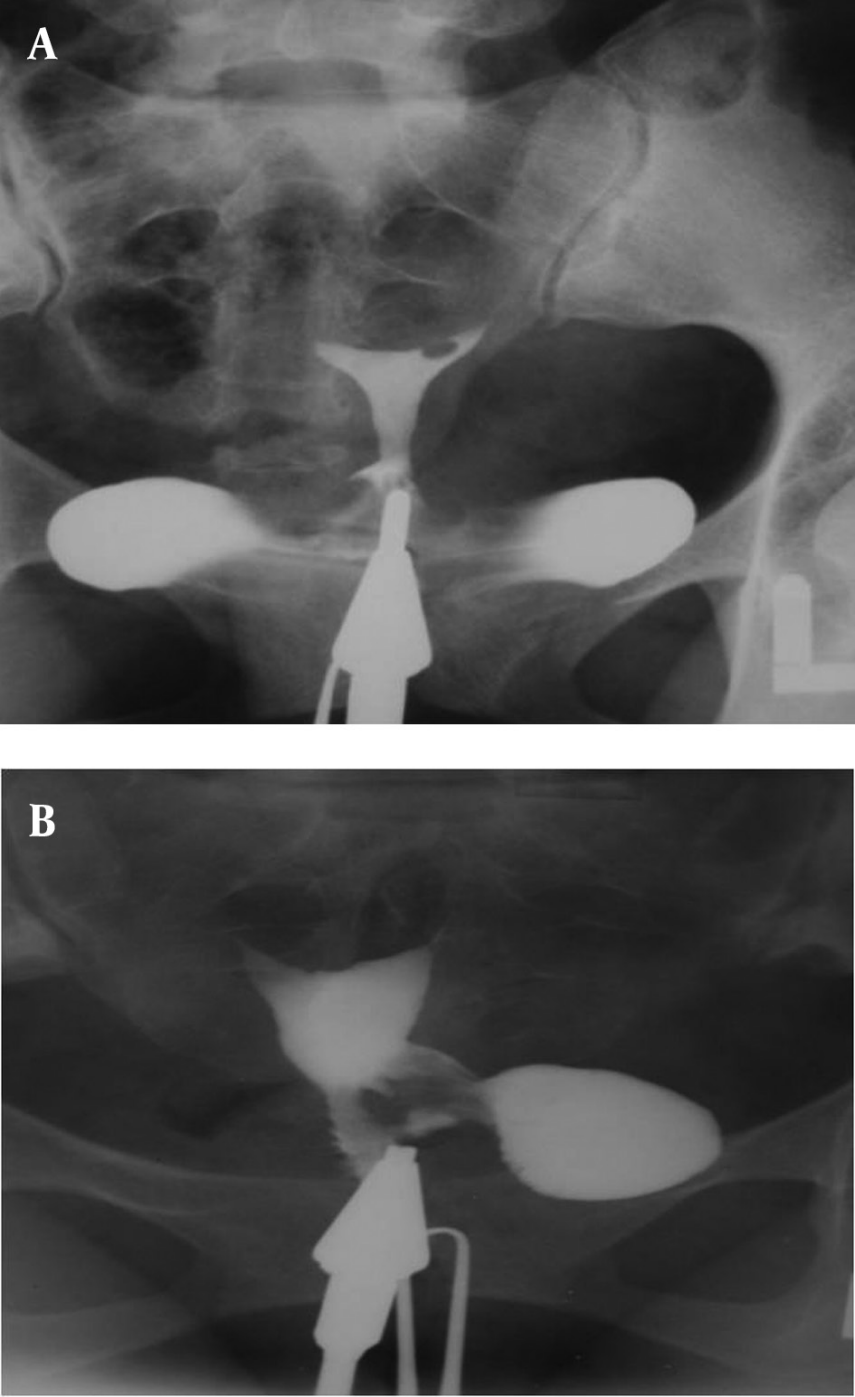
Figure 13. A, A 30-year-old woman with history of four cesarean sections, bloody urine in menstruation and urine exit through the vagina. HSG shows contrast material passed through the uterine isthmus to the bladder proving a uterovesical fistula. B, A 34-year-old woman with a big pouch secondary to passage of contrast material on the left side of the uterine isthmus (fistula to pelvic soft tissue).

3.10. Multiple cesarean scar defects (Figure 14)

**Figure:**
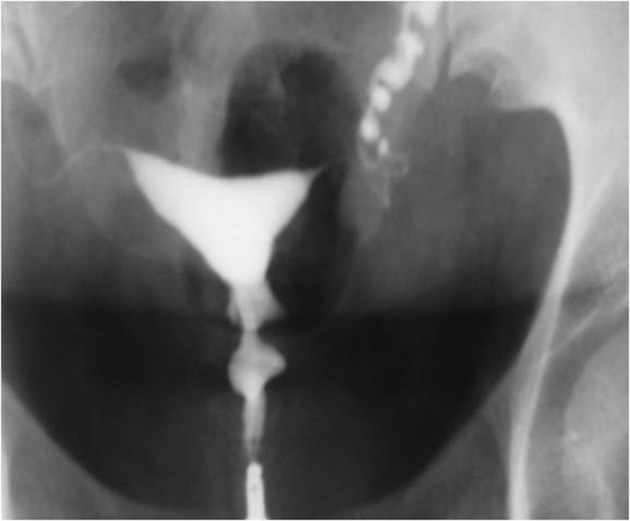
Figure 14. A 34-year-old with multiple cesarean scar defects

In the interpretation of a hysterosalpingogram, awareness of the appearance of the cesarean scar defect is important to avoid misdiagnosis of the scar as underlying pathology or normal variants (8)

**4. Differential diagnoses of cesarean scar defect**

4.1. Prominent cervical glands, small tubular structures arising from the cervical wall, which are typically multiple, bilateral and symmetric unlike the cesarean scar (Figure 15)

**Figure:**
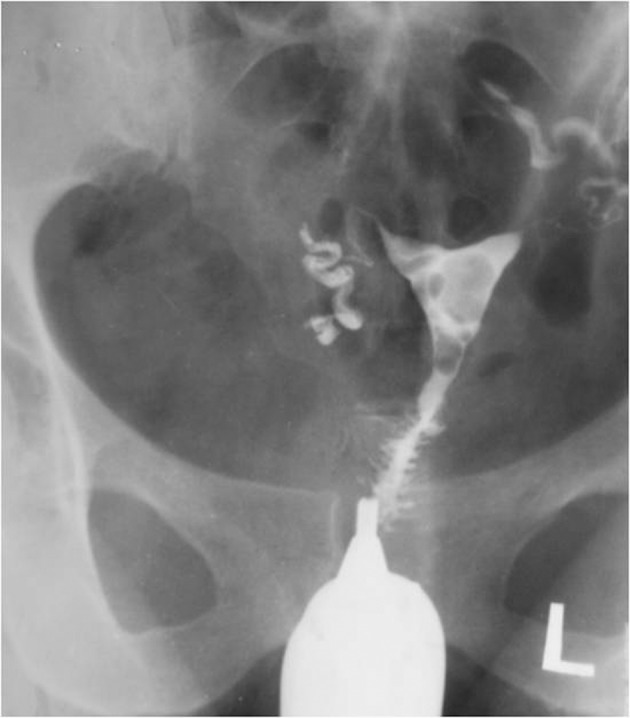
Figure 15. A 32-year-old woman with cesarean section histroy. Diverticula-like structures originating from the cervical wall that corresponds to prominent cervical gland

4.2. Nabothian cyst that is commonly seen in the stroma of the cervix (Figure 16)

**Figure:**
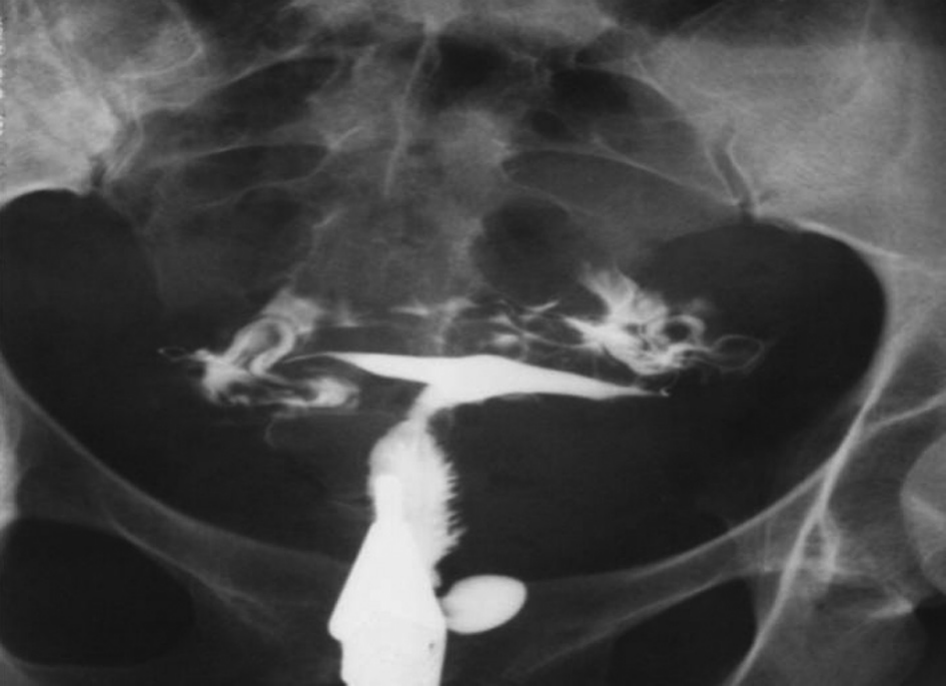
Figure 16. A 28-year-old woman with an outpouch on the left side of the cervical canal proved to be a nabothian cyst (without history of uterine surgery)

4.3. Post myomectomy diverticulum; small outpouching at the resection site other than the cesarean section scar depending on the surgical site. The history may also be helpful.

4.4. Post curettage diverticulum. The patient’s clinical history is important. A previous dilatation and curettage (D&C) history is helpful (Figure 17).

**Figure:**
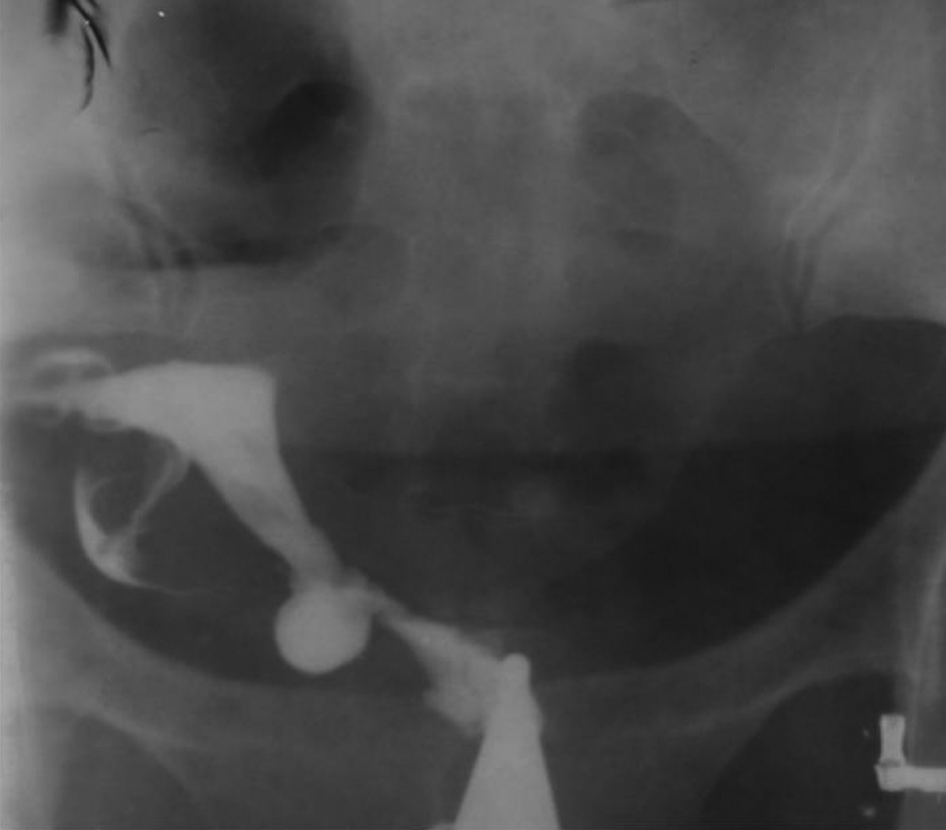
Figure 17. A 28-year-old lady without a history of cesarean section with a pouch on the right side of the uterine isthmus due to uterine curettage

4.5. Congenital cervical diverticula. If the patient has no surgical history, it is helpful in the differential diagnosis (Figure 18).

**Figure:**
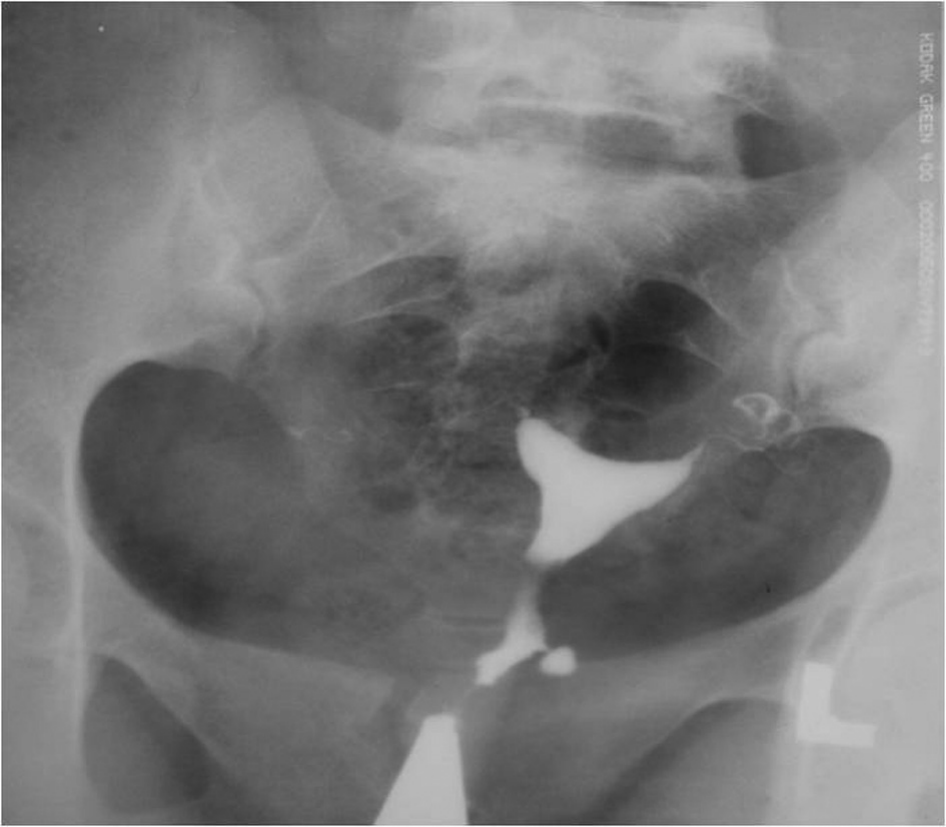
Figure 18. HSG shows small congenital diverticula on the left side of the uterine isthmus in a patient without a history of uterine surgery.

4.6. Focal adenomyosis: Ingrowing of the endometrial tissue into the myometrium with adjacent smooth muscle hyperplasia. It is seen in HSG as fine channels extended perpendicular to the uterine cavity ending in small diverticulum-like structures; focal adenomyosis are multiple and smaller than the cesarean section scar and accompany uterine enlargement, while cesarean section scars are usually larger and single (Figure 19).

**Figure:**
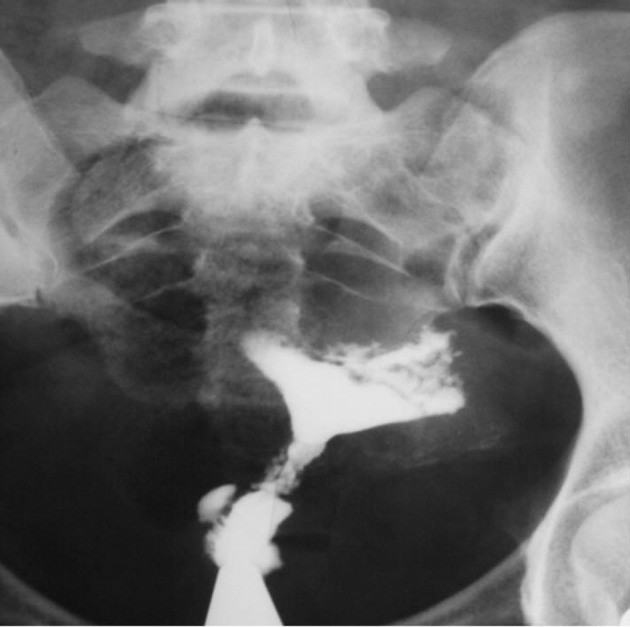
Figure 19. HSG shows honey comb appearance due to localized accumulation of contrast material in the fundal portion and a diverticula in the region of the cervix which may be confused with cesarean scar.

4.7. Tuberculosis: No cesarian section history and obstruction of the fallopian tubes are helpful (Figure 20)

**Figure:**
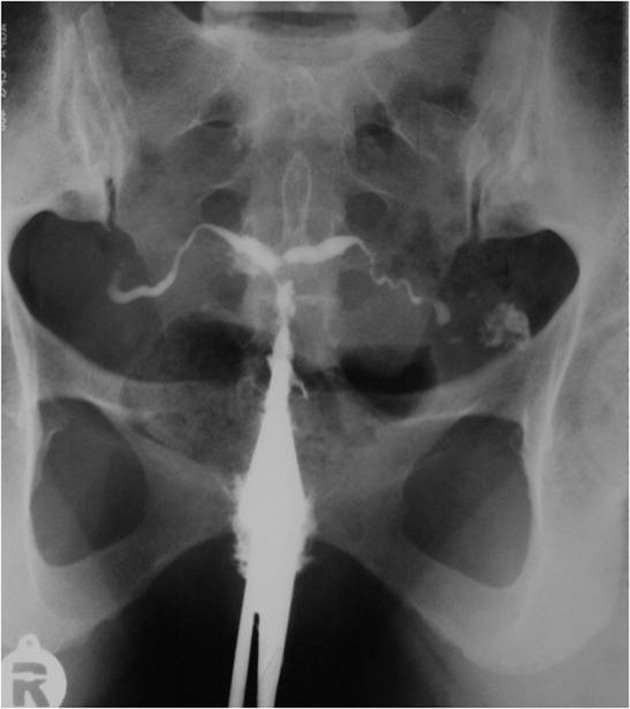
Figure 20. A 28-year-old lady with an irregular cervical canal due to TB infection that may be confused with a cesarean scar. Additionally, there is a typical feature of TB in the uterine cavity and fallopian tubes.
